# Size-dependent Curie temperature of Ni nanoparticles from spin-lattice dynamics simulations

**DOI:** 10.1038/s41598-024-73129-w

**Published:** 2024-09-24

**Authors:** Gonzalo dos Santos, Herbert M. Urbassek, Eduardo M. Bringa

**Affiliations:** 1 CONICET and Facultad de Ingeniería, Universidad de Mendoza, 5500 Mendoza, Argentina; 2 Physics Department, University Kaiserslautern-Landau, Erwin-Schrödinger-Straße, 67663 Kaiserslautern, Germany; 3 Centro de Nanotecnología Aplicada, Facultad de Ciencias, Universidad Mayor, 8580745 Santiago, Chile

**Keywords:** Nickel, Magnetization, Molecular dynamics, Spin dynamics, Nanoparticles, Curie temperature, Atomistic models, Phase transitions and critical phenomena

## Abstract

The magnetic properties of Ni nanoparticles (NPs) with diameter *D* are investigated using spin-lattice dynamics (SLD) simulations. Using exchange interactions fitted to ab-initio results we obtain a Curie temperature, $T_c$, similar, but lower, than experiments. In order to reproduce quantitatively the bulk Curie temperature and the experimental results, the exchange energy has to be increased by 25% compared to the ab-initio value. During the simulated time, Ni NPs remain ferromagnetic down to the smallest sizes investigated here, containing around 500 atoms. The average magnetic moment of the NPs is slightly smaller than that determined experimentally. By considering a core-shell model for NPs, in which the shell atoms are assigned a larger magnetic moment, this discrepancy can be removed. $T_c$ is lower for a moving lattice than for a frozen lattice, as expected, but this difference decreases with NP size because smaller NPs include higher surface disorder which dominates the transition. For NPs, $T_c$ decreases with the NP diameter *D* by at most 10% at $D=2$ nm, in agreement with several experiments, and unlike some modeling or theoretical scaling results which predict a considerably larger decrease. The decrease of $T_c$ is well described by finite-size scaling models, with a critical exponent that depends on the SLD settings for a frozen or moving lattice, and also depends on the procedure for determining $T_c$. Extrapolating the inverse of the magnetization as function of temperature near $T_c$ gives a lower $T_c$ than the maximum of the susceptibility.

## Introduction

In addition to the interest in fundamental research, magnetic nanoparticles (NPs) have attracted attention in a wide range of applications^1,2^. Thus, they found medical uses for magnetic hyperthermia therapy, in particular for the treatment of cancer^3^, and other biomedical applications^4–6^ such as targeted drug delivery^7–9^. Data storage and sensing provide further examples of their existing or anticipated uses.

Besides Fe and Co, Ni is the only element that is ferromagnetic at room temperature. Therefore, the magnetic properties of Ni NPs have been investigated in detail. Small NPs, with a diameter *D* around 3 nm, embedded in silica glass were studied by experiment and simulation^10,11^. He et al.^12,13^ synthesized clusters of pure Ni nanoparticles, revealing significant size and shape effects on their magnetic properties, including saturation magnetization, coercivity, and Curie temperature. In a similar line, Wang et al.^14^ studied clusters of silica-coated Ni NPs focusing on the size dependence of the Curie temperature and its scaling behavior. Ishizaki et al. studied surface-oxidized Ni NP clusters, finding a large size-dependence of the saturation magnetization^15^. Experimental studies on isolated Ni NPs are scarce, but Billas et al.^16^ studied the magnetic properties of small Ni clusters up to around 500 atoms, and Nepijko et al.^17^ studied the size dependence of the Curie temperature in samples of separated Ni NPs from tens to hundreds of nanometers in size.

The theoretical study of NP magnetism is often based on classical spin models and Monte Carlo (MC) simulations^18,19^. NPs with an fcc Fe core and Ni shell were recently simulated^20^, as well as NiCo NPs^21^. Experiments on magnetite NPs display a decrease in Curie temperature of more than 50% as their size decreases, and this behavior was qualitatively explained by MC simulations^22,23^. However, recently molecular dynamics simulations were performed, in which the classical dynamics of the atoms is coupled to the dynamics of the spin system^24,25^; such simulations are known as atomistic spin-lattice dynamics (SLD). Such SLD simulations were applied successfully to study Fe NPs^26,27^ and to explore their temperature-dependent magnetic properties.

In the present study, we use SLD to study Ni NPs, which have received comparatively less attention than Fe NPs. By comparing our results to selected experiments, we are able to elucidate in particular the influence of the NP diameter *D* on the magnetic properties, such as the Curie temperature and the average magnetic moment, which are challenging to obtain under simpler approaches such as the mean-field approximation^28^.

## Methods

### Simulation methods

The calculations are performed in the software LAMMPS^29^. The time step used is 1 fs. The atoms interact through the potential developed by Bonny et al.^30^ which is of the embedded-atom-method (EAM) form.

**Fig. 1:**
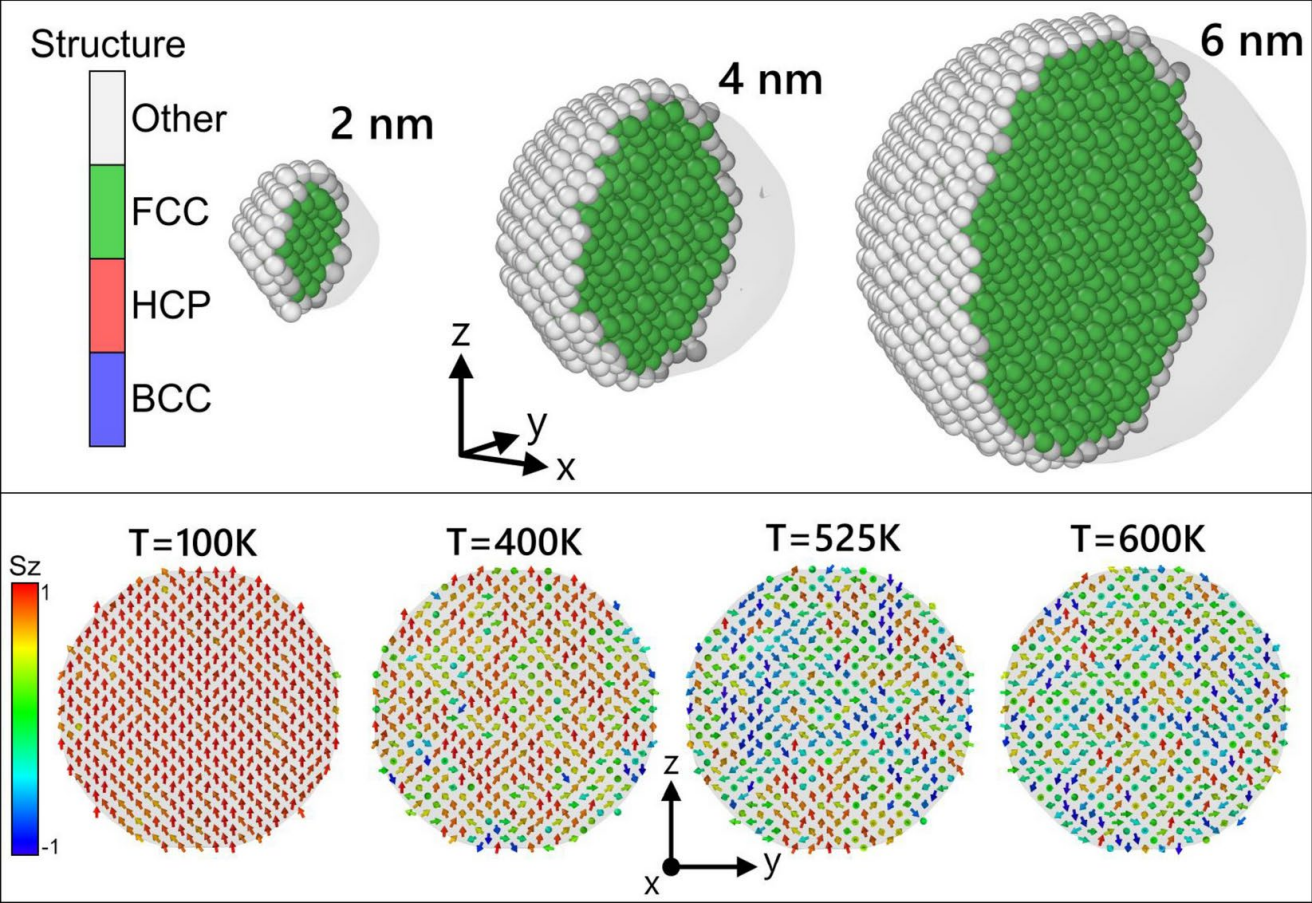
Top panel: Structure types as identified by PTM with rmsd = 0.1^70^ in OVITO^71^, for 3 different NP sizes at 500 K, close to $T_c$, for the final step of the simulations within the moving-lattice approach. Surface atoms are identified as “Other” by PTM due to their low coordination. Shaded regions represent the surface mesh constructed with OVITO. Bottom panel: Typical spins configuration for a 6-nm NP at different temperatures under the moving-lattice approach. Only a 0.5 nm thick slab at the center of the NP is shown. Spins are shown as arrows colored according to their orientation along the *z* axis, indicating clear increase of spin-orientation disorder with temperature.

We consider spherical NPs with a diameter *D* containing *N* Ni atoms. Diameters between 2 and 24 nm are investigated. The spheres are obtained by cutting them out from a fcc Ni crystal with lattice constant $a_0= 0.3524$ nm, giving a nearest-neighbor distance of 0.2492 nm. The number of atoms for the 2, 4, 6 and 24 nm NPs are 381, 3055, 10185 and 661509, respectively. All NPs maintained their fcc structure even at high temperatures as exemplified in the snapshots of Fig. 1. Bulk simulations were carried out for cubic systems with $20 \times 20 \times 20$ fcc cells with periodic boundary conditions.

In SLD, each atom *i* is endowed with a classical spin vector ${\varvec{s}}_i$ of unit length. Initially, each spin points in [001] direction, such that the NPs are fully magnetized. We denote as the magnetization *M* of the NP the average magnitude of the spin,

1 $$\begin{aligned} M= \frac{1}{N} \left| \sum _i {\varvec{s}}_i \right| . \end{aligned}$$

Thus, at temperature $T=0$, it is $M=1$.

Each atom *i* carries a local magnetic moment of size $\mu$ and direction ${\varvec{s}}_i$. In a Ni bulk system, it is $\mu = 0.6 \mu _\textrm{B}$^31^. The average magnetic moment is, thus, given by

2 $$\begin{aligned} \langle \mu \rangle = \mu M. \end{aligned}$$

We assume that all atoms carry the same magnetic moment as in bulk Ni, independently of their location in the NP, which can include lower surface coordination, and a larger atomic volume. However, we have also performed some SLD calculations for a core-shell model in the spirit of Ref.^27^ to account for this variation in the magnetic moment of the surface atoms. For this set of simulations, atoms in the outermost layer of the NP surface have a different magnetic moment, $\mu _s$, than atoms in the core of the NP, $\mu _c$. We chose $\mu _s=0.75~\mu _\textrm{B}$, while $\mu _c=0.6\mu _\textrm{B}$ as for bulk Ni. This particular choice of magnetic moments is discussed below in the Results section. The average magnetic moment in this case is then given by

3 $$\begin{aligned} \langle \mu \rangle = \frac{1}{N} \left( \mu _c \left| \sum _{i=1}^{N_c} {\varvec{s}}_i \right| + \mu _s \left| \sum _{j=1}^{N_s} {\varvec{s}}_j \right| \right) , \end{aligned}$$

where $N_c$ ($N_s$) is the number of atoms in the core (shell).

The Hamiltonian governing the simulations is the given by

4 $$\begin{aligned} \mathcal {H} = \sum _{i=1}^{N} \frac{\left| \varvec{p}_i \right| ^2}{2m_i} + \frac{1}{2} \sum _{i,j,i\ne j}^{N} V(r_{ij})- \frac{1}{2} \sum _{i,j,i\ne j}^{N} J(r_{ij}) \varvec{s}_i\cdot \varvec{s}_j + \mathcal {H}_\textrm{cubic}. \end{aligned}$$

The first two terms represent the kinetic energy and the interatomic potential of the atoms, respectively. The last two terms account for the magnetic contributions to the total energy; they are the Heisenberg Hamiltonian describing the exchange interaction between spins and the magneto-crystalline anisotropy. The latter term is given by

5 $$\begin{aligned} \begin{aligned} H_\textrm{cubic}&= \sum _{i=1}^{N} K_1 [ \left( \varvec{s}_i \cdot \varvec{n}_1\right) ^2 \left( \varvec{s}_i \cdot \varvec{n}_2\right) ^2 + \left( \varvec{s}_i \cdot \varvec{n}_2\right) ^2 \left( \varvec{s}_i \cdot \varvec{n}_3\right) ^2 \\&\quad + \left( \varvec{s}_i \cdot \varvec{n}_1\right) ^2 \left( \varvec{s}_i \cdot \varvec{n}_3\right) ^2 ] + K_2 \left( \varvec{s}_i \cdot \varvec{n}_1\right) ^2 \left( \varvec{s}_i \cdot \varvec{n}_2\right) ^2 \left( \varvec{s}_i \cdot \varvec{n}_3\right) ^2, \end{aligned} \end{aligned}$$

with the unit vectors $\varvec{n}_j$ ($j=1$, 2, 3) along the cubic axes of the crystallite. Further details on our SLD approach can be found in Refs.^26,32^.

In the SLD scheme, the interaction between spins may change the spin direction and also influence the atomic motion. The main contribution is given by the distance-dependent exchange interaction between spins *i* and *j*, $-J(r_{ij}) {\varvec{s}}_i \cdot {\varvec{s}}_j$, where $r_{ij}$ is the distance between atoms *i* and *j*. We use the most recent values provided by^33^, Table I, which give a nearest-neighbor exchange interaction of $J=7.35$ meV. Previous calculations resulted in smaller values, close to 5.71 meV, see^28^, Table I, similar to the values reported in Refs.^34^ and^35^. Even lower values of $J=4.35$ meV can also be found in the literature^36^.

Recently, $J=8.6$ meV was employed to simulate Ni NPs, using only nearest-neighbor interactions^21^.

The spatial dependence of *J*(*r*) is assumed to be of a Bethe-Slater form and we set the cutoff distance at $$r_\textrm{cut}=0.4$$ nm which is a bit further than the second nearest-neighbor distance for the perfect fcc lattice used. In addition, we consider the cubic magnetic anisotropy, whose parameters have been set to $$K_1 =0.34$$$$\upmu$$eV/atom and $$K_2 =0.14$$$$\upmu$$eV/atom^37^.

We note that in the LAMMPS software, the value of *J* has to be multiplied by a factor of two to guarantee the correct counting of the atom pairs in the Heisenberg Hamiltonian, as discussed in the review article by Szilva et al.^38^. This correction was also recently employed by Nieves et al.^39^.

For the frozen lattice approximation we use the same lattice parameter at all temperatures, as it is usually assumed in atomistic spin simulations of nanoparticles^18,31^.

For the moving lattice approximation, the NPs are relaxed during 10 ps, which according to our runs is long enough to ensure that surface relaxation leads to zero global stress. For the bulk moving lattice simulations, the lattice parameter is changed to account for thermal expansion, in order to obtain a global zero pressure for the NP. Spin evolution in LAMMPS does not include a barostat^29,32^, so the relaxation to zero pressure is carried out in two steps: (i) an initial relaxation with a lattice barostat to achieve slightly tensile stress, and (ii) further relaxation with spin integration for an NVE ensemble with Langevin thermostats for both the lattice and the spins.

We use Langevin thermostats to equilibrate our NPs at the desired temperatures for a time of 0.3 ns. After this time, the magnetic properties are measured as an average over 0.2 ns. A Gilbert damping of 0.02 was used^40,41^.

There are several alternative approaches to determine $T_c$^42^. However, we note that the magnetization in NPs is non-zero above $T_c$, due to remaining local magnetic correlations^26,31^. This has also been observed for Ni thin films^43^, and nanowires^44^. The magnetic correlation length diverges at $T_c$ for a bulk system, but it cannot surpass *D* for finite-size systems of size *D*. As a result, for small NPs the critical behavior near $T_c$ is smeared out and this makes difficult the determination of $T_c$^19^. Using the maximum of *dM*/*dT* might underestimate $T_c$^31^, and the maximum susceptibility would provide a better estimate. For this paper we rely on the peak of the susceptibility, defined as the standard deviation of the magnetization, but also include a comparison with another often used method, which considers the intersection of an interpolation of 1/*M*(*T*) with the zero magnetization axis, as it has already been used to obtain $T_c$ for Ni NPs^13^.

The magnetic susceptibility $\chi$ is calculated from the fluctuations of the total magnetization as

6 $$\begin{aligned} \chi \propto \langle M^2 \rangle - \langle M \rangle ^2. \end{aligned}$$

Sometimes, the experimentally measured susceptibility is used to obtain the Curie temperature^45^.

### Scaling of the critical temperature with NP size

In the seminal work by Fisher and Barber^46^ on the phase transition in finite-size Ising systems, they found a scaling of the form:

7 $$\begin{aligned} T_c^D = T_c^\infty \left[ 1- \left( \frac{D_0}{D} \right) ^\lambda \right] , \end{aligned}$$

where $T_c^\infty$ is the Curie temperature of the bulk, $\lambda$ is the shift exponent, $D_0$ is some characteristic size, and *D* is the size scale for the system. $\lambda \le \nu ^{-1}$, with $\nu$ the critical exponent for the correlation length $\zeta \sim D_0 \epsilon ^{-\nu }$, for $\epsilon = 1 - T_c^D / T_c^\infty$. In the initial finite-size scaling studies^46^ and some later work^47,48^, $D_0$ was only associated with some general characteristic length scale of the system. In several studies, $D_0$ has been associated with the lattice parameter of the crystal^14,43^, and it has also been related to the range of spin interactions^49^. Experimental fits often find $D_0$ in the range 1–2 nm which is significantly larger than the lattice constant or the nearest-neighbor distance often used for spin interactions^50^.

This proposed scaling has been observed for many magnetic systems, including experiments and spin dynamics simulations for nanocrystalline FePt^43^, spin dynamics of Co and Gd NPs^19^, MC simulation of Gd NPs^31^. All these cases find exponents $\lambda$ in the range of 1.1–1.6, and $D_0$ around 1 nm. Experiments for Ni nanowires obtained $\lambda =1.3$^51^ and $\lambda =0.94$^44^.

Bertoldi et al.^52^ calculated the Curie temperature $T_c^N$ of a finite-size Ising spin system containing *N* spins and found a power-law decrease as

8 $$\begin{aligned} \left( T_c^N- T_c^\infty \right) \propto ( 1- N^{-\phi }), \end{aligned}$$

where the bulk system has $N=\infty$. The power exponent $\phi$ was found to be around 0.31 for an fcc system like Ni. Given that $N\propto D^3$, this would result in a value of $\lambda$ given by $3 \phi$ and hence around 1, consistent with the results for Ising in Ref.^46^.

For a 3D Ising system, with free boundary conditions like a NP, it was found that $\lambda =\nu ^{-1}~\approx 1$^46^. However, for a 3D bulk Ising Hamiltonian^43^, it is $\nu ^{-1} = 1.587$, indicating that the results in^52^ satisfy $\lambda < \nu ^{-1}$. For a 3D bulk Heisenberg Hamiltonian with a bcc lattice and nearest neighbors, $\nu ^{-1}=0.71^{-1}=1.408$^43^. $\lambda$ is expected to be smaller than this estimate $\lambda =\nu ^{-1}$.

Some experiments on single-crystalline bcc Ni films, with thickness of 1–3 nm, obtained a large exponent $\lambda =2.23$^53^. Other experiments for fcc Ni thin films^54^ reported values $\lambda \le 1$, and proposed an alternative scaling equation to avoid an inaccurate description for small system size:

9 $$\begin{aligned} T_c^D = T_c^\infty \left[ 1+ \left( \frac{D_0}{D} \right) ^\lambda \right] ^{-1}. \end{aligned}$$

## Results

### Frozen versus moving lattice

**Fig. 2:**
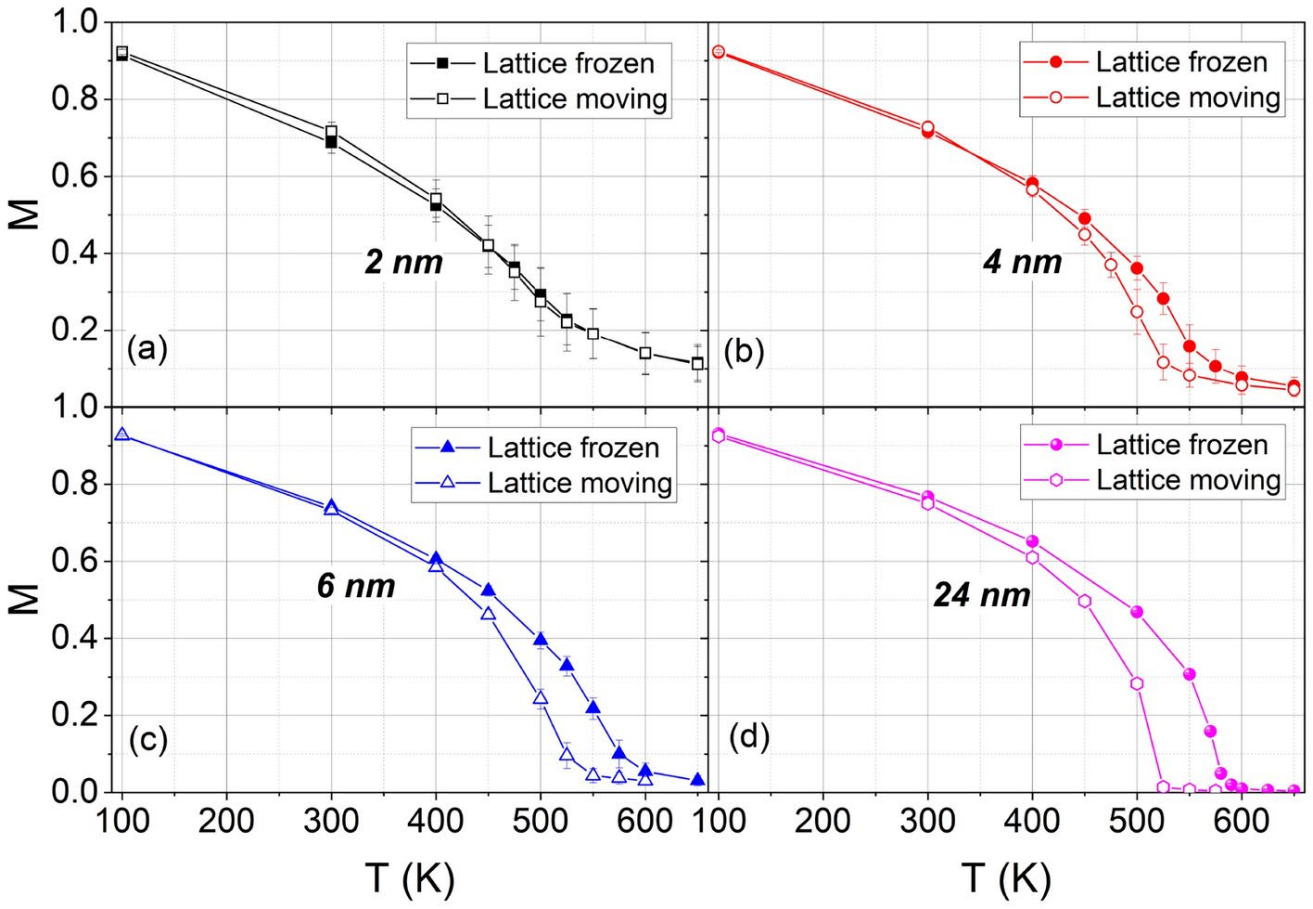
Temperature dependence of the magnetization *M* of Ni NPs with various diameters *D*. Results for the moving lattice as well as for the frozen-lattice approximation are compared.

Figure 2 shows data for the temperature dependence of the magnetization *M* of NPs of various sizes. This figure also shows the effect of the thermal vibrations of the atoms on the magnetization by comparing the results of a full SLD calculation with that of a frozen-lattice approximation. Clearly, the effect of vibrations increases with temperature. Also, the relative importance of vibrations increases with NP size. This was also reported for SLD simulations of Fe NPs^26^. The spin disorder due to the fraction of low-coordinated surface atoms competes with additional disorder due to finite-temperature lattice vibrations. For the smallest NPs studied, $D=2$ nm, the effect of vibrations on the magnetization is negligible because surface disorder, which is also present in the frozen structure, is more relevant than lattice vibrations. A similar figure for bulk samples can be found in Fig. S1 of the Supplementary Material. Typical spin configurations at different temperatures can be observed in the bottom panel of Fig. 1.

**Fig. 3:**
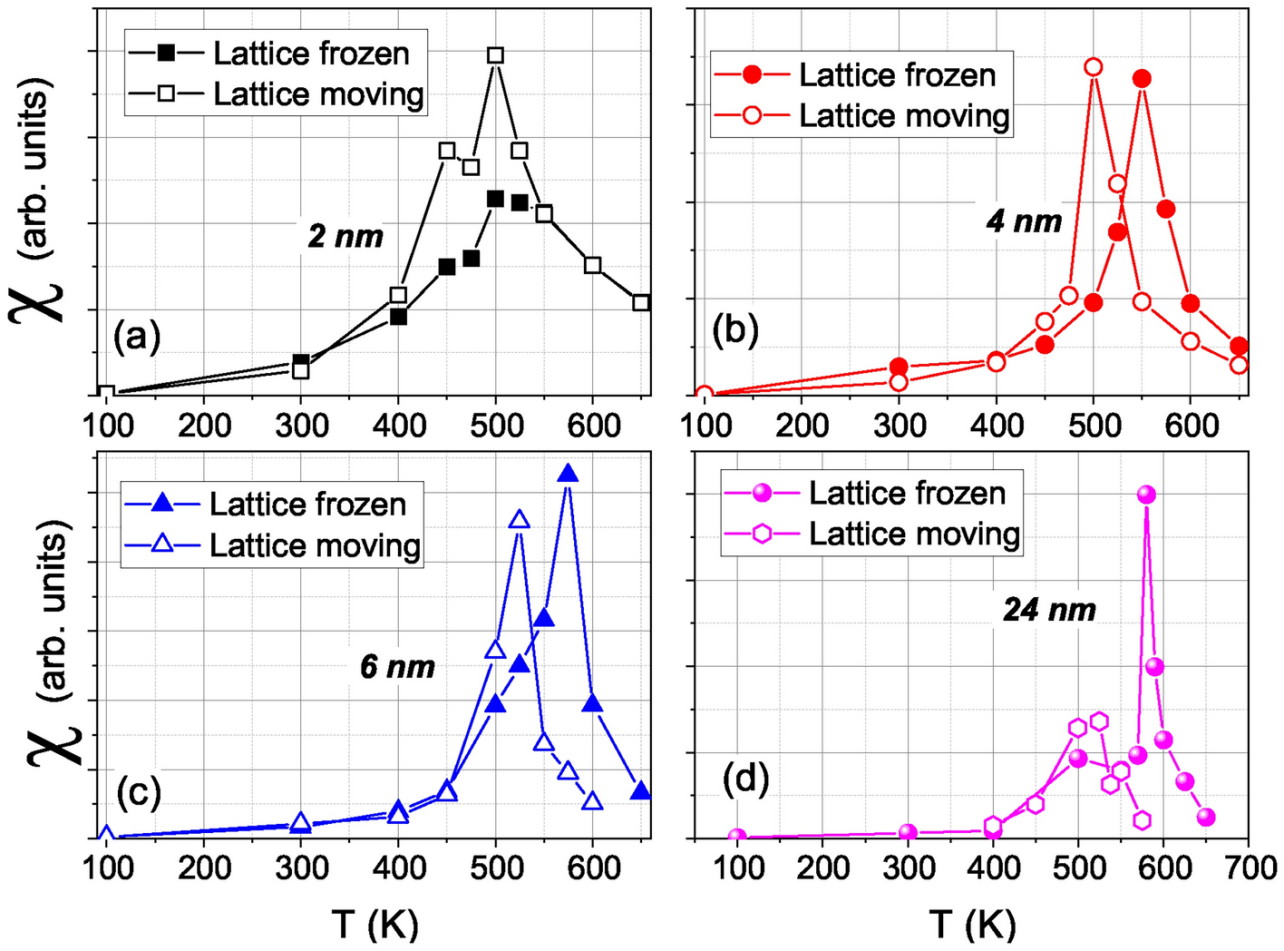
Temperature dependence of the susceptibility $\chi$ of Ni NPs with various diameters *D*. Results for the moving lattice as well as for the frozen-lattice approximation are compared.

Simulation results for the magnetic susceptibility $\chi$ are shown in (Fig. 3). Note that $\chi$ has its maximum at the Curie temperature. For the susceptibility, the influence of lattice vibrations appears to be even stronger than for the magnetization, Fig. 2, and persists up to the largest NPs investigated, $D=24$ nm.

**Fig. 4:**
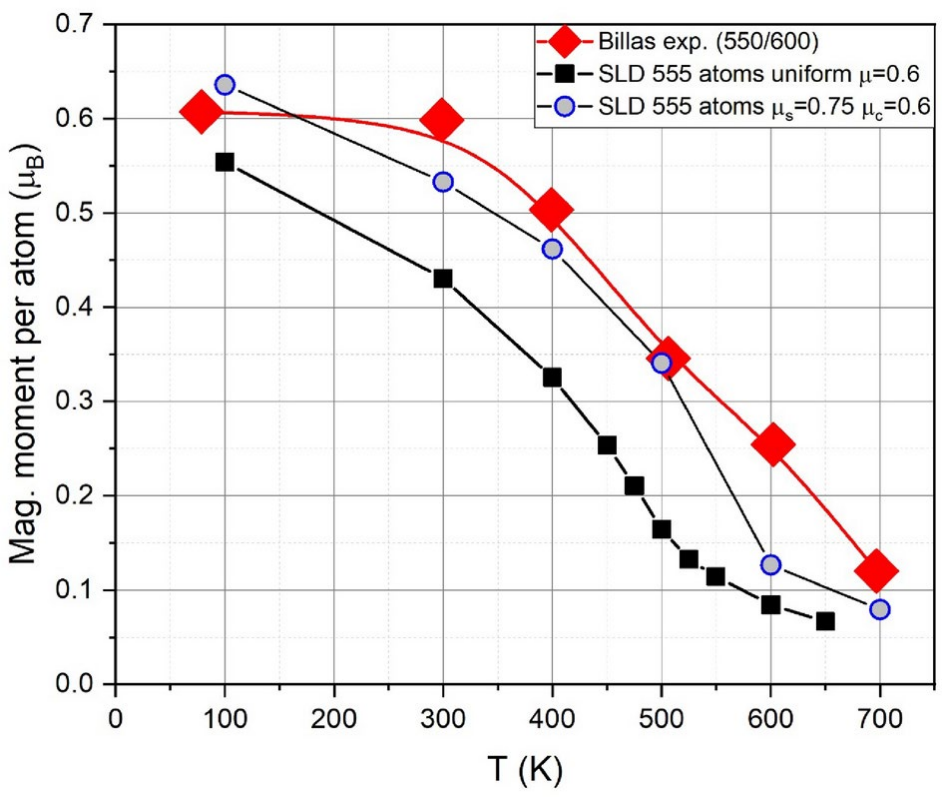
Temperature dependence of the average magnetic moment per atom $\langle \mu \rangle$ of Ni NPs with diameter $D=2.3$ nm, containing 555 atoms. SLD results for a uniform model are compared to those for a core-shell model and to experimental data by Billas et al.^16^. SLD was performed for a moving lattice.

Experimental data for the temperature dependence of the magnetic moment per atom, $\langle \mu \rangle$, are available for NPs containing 550–600 atoms^16^. We compare these data in Fig. 4 with our simulation results for NPs of diameter $D=2.3$ nm (containing 555 atoms). While the shape of the theoretical curve agrees well with the experiment, the theoretical magnetic moments are too small compared to the experiment. We propose that this may be caused by a surface effect, since the magnetic properties of surface atoms may be assumed to be changed with respect to bulk atoms^27^. Their magnetic moment will be increased due to the so-called magneto-volume effect^55,56^, and for a free Ni atom, it is $\mu = 1.0~\mu _\textrm{B}$^16^. Unfortunately, detailed calculations of the increase for surface atoms appear not to be available.

We have employed a core-shell model to emulate surface effects on the magnetic moment^22,27^, assuming a magnetic moment of $\mu _s=0.75~\mu _B$ for the surface atoms (lower than that of a free Ni atom), and $\mu _b=0.6~\mu _B$ for core atoms, as stated in the methods section, according to Ref.^16^. Figure 4 shows that the core-shell model gives a closer agreement of the NP magnetization (calculated with Eq. (3)) with the experimental data. We note, however, that also the exchange interaction *J* between shell atoms, and between shell and bulk atoms, may change from its value between bulk atoms. Core-shell interaction strength was parametrically varied to study its influence on the magnetization of a model hcp NP described with a nearest neighbor Ising Hamiltonian^57^, but those findings cannot be easily extrapolated to our system. We are not aware of any experiments nor ab-initio studies quantifying this effect for Ni, and such interaction changes are not considered in the present study.

**Fig. 5:**
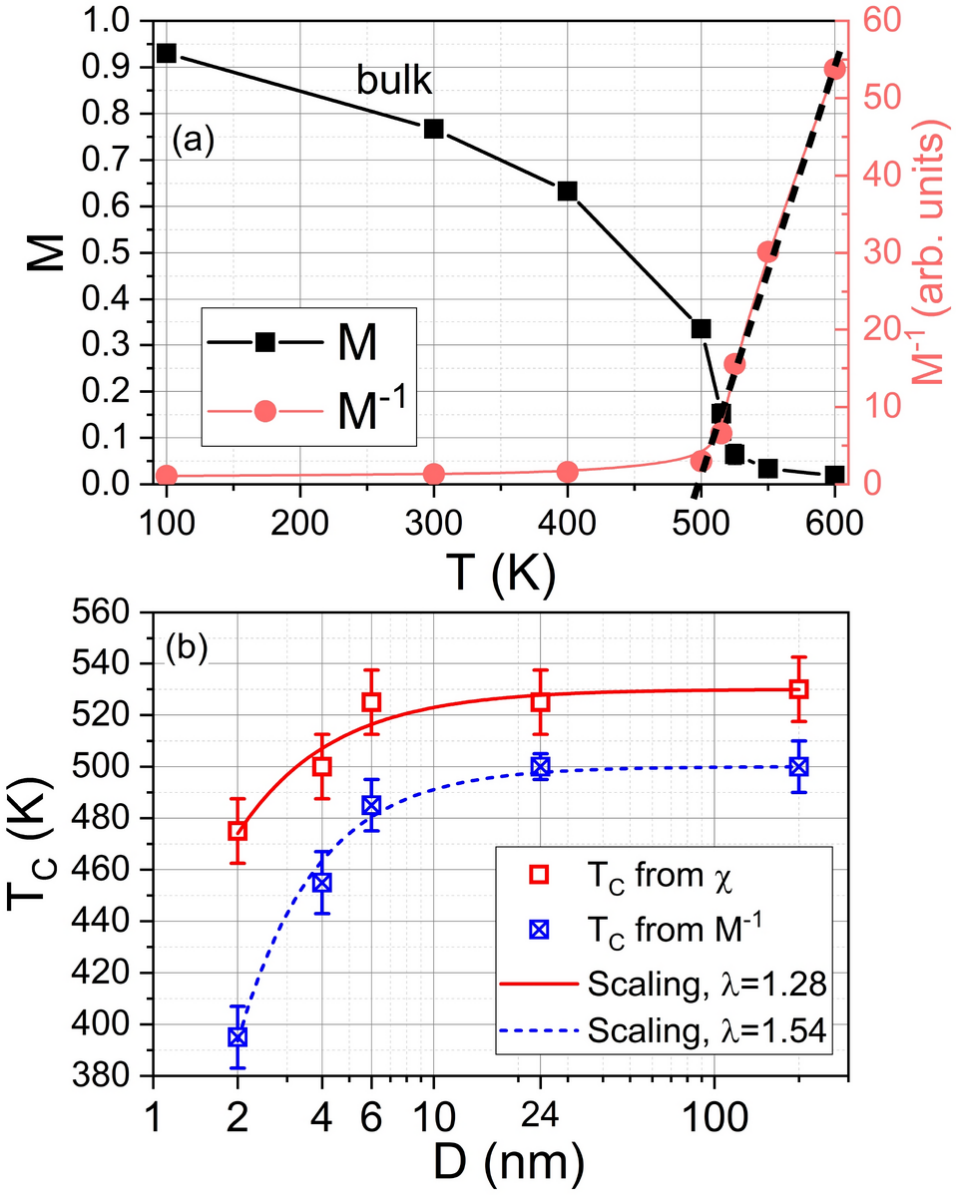
(**a**) $M-T$ and $M^{-1}-T$ curves for the bulk sample under the moving-lattice approach. (**b**) Curie temperature vs NP size (log scale) obtained from the maximum of the susceptibility and from $M^{-1}$ extrapolation. Lines represent fits to the scaling relation given by Eq. (7). The points at 200 nm represent the corresponding bulk results.

Figure 5 shows the Curie temperature $T_c$ as a function of the Ni NP diameter *D*, also including bulk results, for moving lattice simulations. Two different approaches are employed to determine $T_c$: the maximum in the susceptibility and the method of inverse magnetization^13^. The scaling fits using Eq. (7) provide different scaling exponents and characteristic lengths, despite being obtained from the same magnetization curves.

**Fig. 6:**
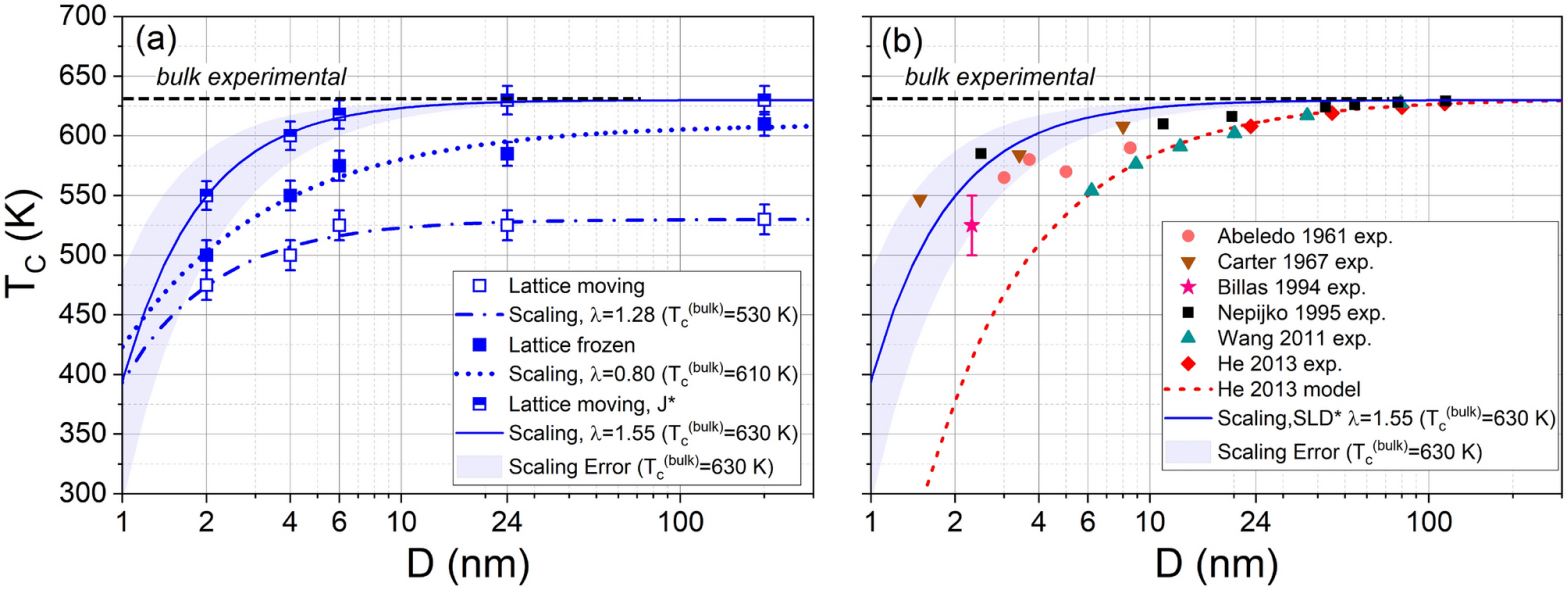
Dependence of the Curie temperature $T_c$ on the diameter *D* of Ni NPs. (**a**) Results for the moving lattice as well as for the frozen-lattice approximation. The points at 200 nm represent the bulk results for the corresponding simulations. Lines represent fits to the scaling relation given by Eq. (7), with parameters given in (Table 1). The results labeled as “Lattice moving, $J^*$” correspond to the simulations using the exchange function enhanced by a factor of 1.25 as discussed in the text. (**b**) Experimental data from several sources included as symbols: He et al.^13^, Wang et al.^14^, Billas et al.^16^, Abeledo et al.^72^, Nepijko et al.^17^, Carter et al.^73^. The black line shows the theoretical model reported by He et al. to fit their data^12^. The fit for the SLD moving lattice results shown in panel (**a**) is included as solid blue line; the shaded region indicates the error in the scaling exponent. Other lines indicate the scaling exponents as listed in Table 1.

Finally, Fig. 6a displays the Curie temperatures of the Ni NPs from the susceptibility maxima in Fig. 3 for both moving and frozen lattice approaches. The clear influence of lattice vibrations on the Curie temperature was already noted above in the discussion of Fig. 3. It demonstrates the main advantage of SLD calculations—i.e., the coupling of spin dynamics and atomic motion. Fe NPs also showed such a large difference between moving and frozen, and it was anticipated that this would modify $T_c$^26^. One might argue that thermal lattice expansion due to surface effects might be significant near the Curie temperature, leading to a decrease in magnetic exchange interactions, but the pair correlation function, shown in Fig. S2 of the Supplementary Material, shows negligible change in the average nearest-neighbor distance when going from 100 K to 525 K, similarly to what happened for Fe near $T_c$^26^. We also studied the influence of the magneto-crystalline anisotropy on the magnetization curves and on $T_c$, by running a simulation with an anisotropy constant ($K_1$) 10 times higher than the original one. The results are shown in Fig. S3 of the Supplementary Material, where a negligible influence of anisotropy is observed.

Note that our calculated bulk Curie temperatures from SLD are smaller than the experimental value of 630 K^12^, see Fig. 5 and Fig. S1 in the Supplementary Material; this is a known feature of the ab-initio calculated exchange interactions^28^. In mean-field models and MC simulations of the Heisenberg Hamiltonian $T_c \propto z J$^31^, where *z* is the coordination number. For a NP the mean *z* value will decrease, causing a decrease in $T_c$. For bulk system, $z=12$ for fcc, and we would have to increase the magnitude of the ab-initio exchange interactions in order to match experimental values. This would mean multiplying the current *J* by $T_{c,exp}/T_{c,SLD}=630/530=1.19$, to obtain the correct $T_c$ from experiments. This would give a scaled-up value of 8.75 meV for nearest neighbors.

Recent MC simulations for bulk Ni obtained a bulk $T_c=623$ K, using $J=8.51$ meV^20^. In addition, MC simulations for a 6 nm Ni NP using nearest-neighbor exchange $J=8.6$ meV led to $T_c=626$ K^21^. Instead of using 1.19 as a multiplier, we find that an exchange function given by $J^*(r)=1.25 J_{DFT}(r)$, which gives at NN distance a value of around 9.19 meV, leads to the correct bulk $T_c$, in good agreement with the above MC simulations. SLD results for a moving lattice with this higher exchange function $J^*(r)$ are also included in Fig. 6a.

The mean-field approximation for the nearest-neighbor fcc Heisenberg Hamiltonian gives $k_B T_c =(1/3)zJ=3.16 J$ for the bulk fcc system^31^. MC simulations for the same system give $k_B T_c =0.79(1/3)zJ=2.4964 J$^58^, supporting this linear scaling between $T_c$ and *J*. Using $J=14$ meV from nearest neighbors gives $T_c$ around 514 K for mean-field and around 405 K for MC. These values are very low compared with the experimental value but in line with the values from^28^, which reported 397 K for the mean field approximation.

Figure 6a shows reasonable agreement of our simulation data with Eq. (7). However, the value of $\lambda$ is below 1 for the frozen lattice. It is likely that, as in the case of^52^, $\lambda < \nu ^{-1}$. Fit parameters are given in Table 1.

**Table 1:** Critical exponents for several experiments and simulations. The critical temperatures for bulk MC simulations, for the Ising model ($k_B T_c = 9.794 J$)^69^ and for the Heisenberg model ($k_B T_c = 3.16 J$)^31^ were obtained using $J=14$ meV. “SLD moving*” indicates simulations with *J*(*r*) enhanced by a factor of 1.25 compared to the ab-initio value.

Data/model	$T_c^{\infty }$ (K)	$D_0$ (nm)	$\lambda$	Ref.
He 2013	630	$0.94 \pm 0.21$	$1.04 \pm 0.07$	^13^
Wang 2011	630	$0.65 \pm 0.10$	$0.94 \pm 0.05$	^14^
Nepijko	630	$0.26 \pm 0.10$	$0.89 \pm 0.08$	^17^
Ising	1590	–	1.587	^43^
Heisenberg	514	–	1.43	^58^
SLD frozen	610	$0.23 \pm 0.10$	$0.80 \pm 0.14$	This work
SLD moving	530	$0.35 \pm 0.17$	$1.28 \pm 0.32$	This work
SLD moving*	630	$0.53 \pm 0.10$	$1.55 \pm 0.20$	This work

The scaling from Eq. (9) was also tried for the frozen lattice, yielding $\lambda =0.88 \pm 0.17$, only slightly larger than for the fit with Eq. (7). The scaling exponent for the moving lattice is $\lambda =1.28$, which is slightly lower than the exponent $\lambda =1.408$ expected from a nearest-neighbor Heisenberg Hamiltonian^58^.

### Comparison with experiments and models

We find only a slight dependence of $T_c$ on NP diameter, with a noticeably decrease only for NP diameters $D \lesssim 6$ nm. Our findings are in excellent agreement with experiments, as shown in Fig. 6b. Experimental error bars are not included, except for Billas et al.^16^. They demonstrate in their Fig. 2a that the moments of Ni clusters containing more than around 300 atoms are already bulk-like. Although the data from Ref.^16^ is sparse, it suggests that $T_c$ does not decrease significantly for small clusters compared to bulk, in contrast to the model predictions in^12^. Our results can also be compared to experimental data for larger NPs with diameters of 20–200 nm^12,13^. At $D= 24$ nm, where our calculations overlap with the experimental NP size, we find good agreement with the experimental result.

Note that the excellent agreement of our results using the exchange function enhanced by a factor of 1.25 (Fig. 6a, half-filled squares) is achieved for the full SLD simulation scheme, i.e. the moving lattice approach. If we had used the frozen lattice approximation, similar to a typical Spin Dynamics simulation, the Curie temperatures obtained would overestimate the experimental values.

Our simulations and the experiments by Billas and coworkers^16^ are for isolated NPs. Experiments by He and coworkers for the larger NPs^12,13^ measured a large collection of agglomerated NPs, which would have different crystal orientations and also dipolar interactions affecting the overall measured magnetization. It is difficult to assess the role of the collection of crystal orientations and dipolar interactions on the global Curie temperature of the ensemble. Nepijko and Wiesendanger^17^, based on a simple lattice model and molecular field theory, argued that $T_c$ would increase as NPs are closer to each other.

Scaling fits to the experimental data for ‘large’ NPs^13^ do not appear to provide a correct extrapolation towards small NP results, as demonstrated by the results for a 6 nm NP^16^, which fall well above those scaling predictions.

From our fits, $D_0 \sim 0.2$–0.5 nm, which is in line with the assumption^13^ that $D_0\sim a_0=0.35$ nm. Assuming that $\lambda =\nu ^{-1}$, the spin correlation length $\zeta$ would be shorter for Ni NPs in this study than for experiments on Ni films, or for hcp ferromagnets like Co. Within the assumption that $D_0$ is related to the range of spin interactions^49^, $D_0 \propto \zeta$, and this would be consistent with a larger value obtained from thin-film experiments, $D_0$ around 1 nm^49^. For the simulations here the range of exchange interactions is $\sim a_0=0.35$ nm.

### Models for the Curie temperature

There are many numerical and analytical models to obtain the Curie temperature versus NP size. Nikolaev and Shipilin^59^ proposed a simple model, where $T_c$ is proportional to the number of exchange bonds (interacting pairs), and this number is reduced by 0.5 for surface atoms, leading to $T_c /T_c^\infty =1 - 3 \delta / D$. $\delta$ is the surface thickness, which would also depend on *r*, and the model adjusts this based on particular experimental results for $\hbox {Fe}_3\hbox {O}_4$ NPs.

Recently, the magnetization of finite Ising systems was evaluated within the numerical *mean spin method* assuming that there is a fraction of non-magnetic atoms occupying a regular lattice, with magnetic atoms interacting to first neighbors^23^. The critical temperature for the ferromagnetic/paramagnetic transition was associated to a percolation transition of the sites occupied by magnetic atoms, i.e. above the percolation transition the average magnetization drops to zero. For finite sizes, a cube with an edge containing $N_a$ atoms was considered, and all edge sites are considered non-magnetic, leading to a decrease of the magnetization with cube size. When this model was applied to thin films instead of NPs, the scaling exponent for Ni thin films was similar to the ones discussed above and gave values of $\lambda$ around 1.5.

There is another model for the size-dependence of $T_c$ for Ni nanoparticles, following a model inspired by the size dependence of the glass transition temperature^60^. The model was assumed to work also for Ni nanowires and requires many parameters, including the specific heat difference between ferromagnetic and paramagnetic phases, surface energies, etc., and predicts a rapid exponential descent of $T_c$, down to around 540 K for a 30 nm Ni NP^60^.

We note that in their experimental work, He and Shi^12^ fit their data to a model borrowed from the description of cluster melting^61,62^; it predicts an exponential decrease of the Curie temperature for small cluster sizes and a complete breakdown of ferromagnetism at finite cluster sizes of around 2 nm. The same model has been used to explain MC simulation results for Fe NPs^18^. Our data are in clear disagreement with this last model. In particular, our calculations show that clusters even as small as 2 nm are ferromagnetic with a Curie temperature less than 10% below the bulk value. This is in agreement with the experiments by Billas et al.^16^ discussed above. Also, in thin Ni films grown on a Cu substrate, it was shown experimentally that for film thicknesses beyond around 4 nm, the Curie temperature saturates^63^.

In a recent MC simulation paper, Dung and Hung^64^ used NP configurations from previous MD simulations^65^, to obtain the Curie temperatures of Ni clusters with sizes of 4.5–6 nm. $T_c$ assumed values between 400 and 500 K, indicating an strong decreased with NP size. The NPs used were not single-crystalline as in our simulations and most experiments, but nanocrystalline, and they also contained hcp clusters besides the fcc phase. This is a consequence of the huge quenching rate with which these NPs were quenched from the molten phase^65^. Furthermore, the authors fitted the value of the exchange interaction to obtain agreement with an extrapolation of experimental data by He and Shi^12^, which was carried out using the ‘melting’ model^12^ mentioned above. These findings are in contrast to experiments for small NPs^16^ and to our SLD results using exchange from ab-initio data.

Although we find a relatively small dependence of $T_c$ with size, this dependence might be larger for NPs with an oxide surface ‘dead layer’, as shown for the saturation magnetization of Ni NPs^15^. Finally, as another possible factor affecting $T_c$, we note that quantum effects will change the *M*(*T*) curve. A quantum Heisenberg model features slightly higher magnetization at temperatures below the Curie temperature^66^. These effects are outside of our classical SLD study, but the value of the Curie temperature is not greatly changed.

## Summary

We studied the ferromagnetic properties of Ni NPs using spin-lattice dynamics (SLD) and found the following features.

1. Using the exchange *J* from ab-initio results^33^, Table I, the Curie temperature, $T_c$, simulated for the bulk, is lower than in experiments. This is similar to previous model^28^ and simulation results for Ni. Increasing this value of *J* by 25% does provide $T_c$ in agreement with experiments, especially considering errors in the simulation and experiments.
2. On the methodological side, the influence of thermal vibrations lowers the magnetization of NPs at high temperatures and shifts the Curie temperature $T_c$ to lower values; this feature is in support of the use of SLD for the study of magnetic phenomena in NPs.
3. The effect of thermal vibrations becomes smaller with decreasing NP size, as surface disorder dominates over thermally induced disorder.
4. The average magnetic moment of the NPs is smaller than that determined experimentally^12^. The discrepancy can be healed by considering a core-shell model for NPs^27^, in which the shell atoms are assigned a larger magnetic moment.
5. Ni NPs remain ferromagnetic down to the smallest sizes investigated here—$D=2$ nm, containing around 500 atoms. This would change for macroscopic time scales, where thermally-induced magnetization flips would result in a measured superparamagnetic state for such small NPs^67^.
6. The Curie temperature $T_c$ decreases with NP diameter *D*. This is well described by scaling models^46,52^, but the scaling exponent depends on whether the lattice is allowed to move or is frozen, and also on the method to determine $T_c$.
7. Scaling fits for experimental results do not necessarily provide reliable extrapolation to small NP diameters.
8. The decrease of $T_c$ with *D* is at most around 10% even for small NPs, with $D \lesssim 2$ nm. This is in agreement with experimental findings^16^, but in disagreement with scaling extrapolations from larger NPs^43^ and with some MC simulations^64^.

In summary, spin lattice dynamics (SLD) simulations can help understanding experimental results for the magnetic behavior of NPs, testing magnetic interaction values and providing alternative scenarios which are difficult to explore with experiments or other simulation methods.

Future simulations might tackle the dynamic behavior of other magnetic nanostructures, including core-shell NPs, nanowires, and thin films, to find their $T_c$.

The dependence of the Curie temperature on NP size can be tuned with the help of surface effects^15,22,27^, engineering different core-shell NPs, and SLD might provide useful guidance to future experiments to optimize magnetic properties. $T_c$ can also be tuned with strain^68^, and this could be accomplished with NPs embedded in a matrix.

## Electronic supplementary material

Below is the link to the electronic supplementary material.

Supplementary Information.

## Data Availability

All data used for this study are contained in this article.
