# Supplementary material for: Size-dependent Curie temperature of Ni nanoparticles from spin-lattice dynamics simulations
Source: Sci Rep. 2024 Sep 24;14:22012. doi: 10.1038/s41598-024-73129-w (PMC11422501; doi:10.1038/s41598-024-73129-w)
Supplement: Supplementary file 1 — Supplementary Information. [file 41598_2024_73129_MOESM1_ESM.pdf]

Size-dependent Curie temperature of Ni nanoparticles from spin-lattice dynamics simulations
Supplementary Material

Gonzalo dos Santos,[1] Herbert M. Urbassek,[2,*] and Eduardo M. Bringa[1,3]

[1]*CONICET and Facultad de Ingeniería, Universidad de Mendoza, Mendoza, 5500 Argentina*
[2]*Physics Department, University Kaiserslautern-Landau, Erwin-Schrödinger-Straße, D-67663 Kaiserslautern, Germany*
[3]*Centro de Nanotecnología Aplicada, Facultad de Ciencias, Universidad Mayor, Santiago, Chile 8580745*

(Dated: August 13, 2024)

Fig. S1 presents our results for bulk Ni for the moving lattice and for the frozen lattice.

Fig. S2 displays the pair correlation function for a 6-nm NP, showing only negligible changes in the average nearest-neighbor distance between 100 K and 525 K.

Fig. S3 shows a comparison of magnetization curves for a 6-nm NP with different anisotropy constants between 400 K and 600K. An increase of the anisotropy constants by a factor of 10 leads to only negligible changes in the magnetization values.

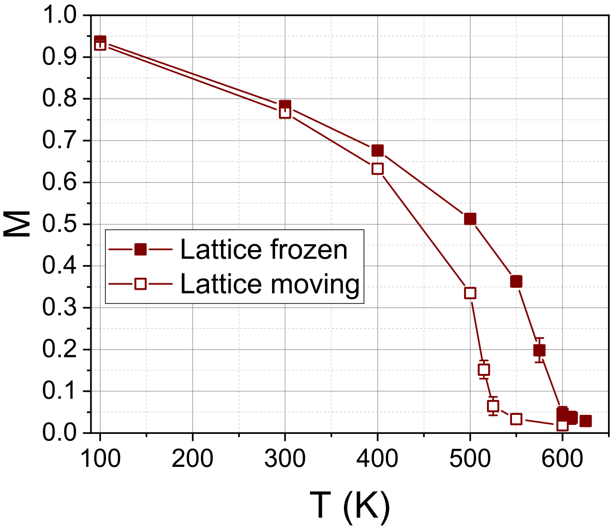

FIG. S1. Temperature dependence of the magnetization M of bulk Ni. Results for the moving lattice are compared to the frozen-lattice approximation.

* urbassek@rhrk.uni-kl.de; http://www.physik.uni-kl.de/urbassek/

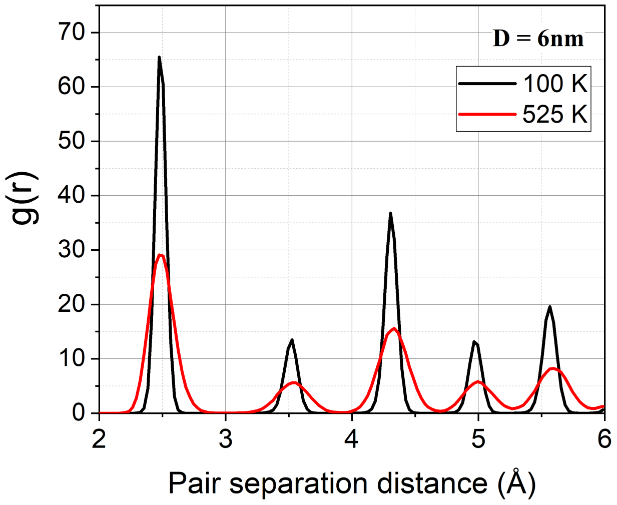

FIG. S2. Pair correlation function $g(r)$ for a Ni NP with diameter $D = 6$ nm, at two different temperatures. Note that the average nearest-neighbor distance, which is crucial for exchange interactions does not change significantly from 100 K to a temperature just below the Curie temperature T_c. $g(r)$ also indicates that the material is solid near T_c. For the frozen lattice, $g(r)$ would be a collection of delta functions at all temperatures, with the first peak at 0.2492 nm, and the second peak at 0.3524 nm.

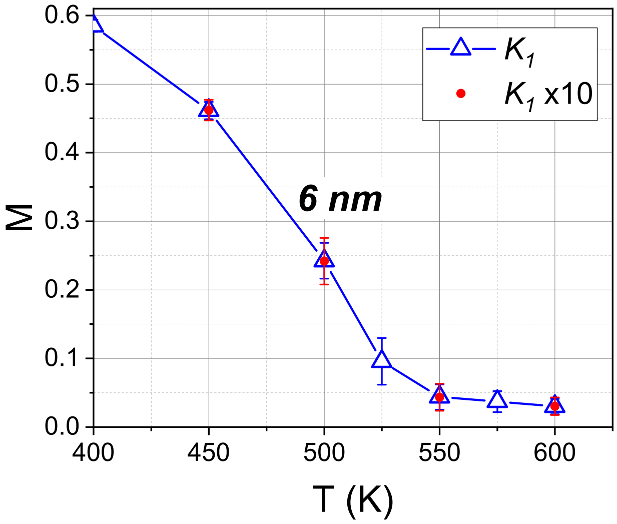

FIG. S3. Temperature dependence of the magnetization M of a 6 nm NP with the original anisotropy constant K_1 and with the anisotropy increased by a factor of 10, using the moving lattice approach.
